# The Four Insurance Commissions

**Published:** 1913-06-14

**Authors:** 


### OUR NATIONAL INSURANCE ACT DEPARTMENT.

### LVI.—The Four Insurance Commissions.

In the forefront of suggested amendments to the National Insurance Act, as drafted by those with wide practical experience of its working, is the abolition of the four separate Insurance Commissions. Yet we understand that the suggestion is not likely to meet with acceptance by the Government. That some means should be devised to prevent the present useless overlapping of administrative machinery and to simplify the duties of those whose work it is to dispense the benefits of the Act is, however, apparent. There is, of course, the co-ordinating Joint Committee of Commissioners, but in spite of this the orders of the separate Commissions are of remarkable dissimilarity in certain respects, and the worst results of the present system have not yet emerged.

### Consequences of Divided Authority.

No better illustration can be given of the difficulties experienced by officials of approved societies through the quadruple control than that of the recent orders issued for the election of representatives of insured persons to serve on the Insurance Committees. Our last article dealt at length with the system adopted by the English Commissioners, and showed how complicated it was. We might have extended our remarks by calling attention to the orders issued by the Scottish and Welsh Commissioners respectively on the same subject. The Irish orders have not yet been issued, but it is a foregone conclusion that they will have features distinguishing them from those already issued. The orders of the three Commissions respecting the election of representatives of insured persons to Insurance Committees that have already been published differ essentially from each other, not only in regard to the dates of nomination and election, but also in regard to the system whereby proportionate representation is to be secured. In such matters as these it is difficult to see that there is any advantage to be gained by separate action. It is essential to the smooth working of the Insurance Act that exceedingly capable men should be elected to serve on the Insurance Committees, and it is only courting disaster to place before the officials of approved societies with members in all four countries of the Union no less than four distinct sets of orders to be complied with as regards the different sections of their members.

### The Allocation of Insured Persons to Panel Doctors.

That service on an Insurance Committee is no sinecure is evident from the present situation of the London Insurance Committee. Owing to the failure of insured persons resident within the administrative County of London to notify their choice of doctors, the Committee is faced with the colossal task of allocating about 400,000 insured persons to doctors on the panel. Five months have expired during which these people could have exercised their choice, but they have been too apathetic to the advantages of the Act to take the trouble of referring to the list of panel doctors and making a choice therefrom.

### The Surplus.

The result is the London Insurance Committee has a surplus of about £34,000 which is due under the provisions of the National Insurance Act to the doctors on the panel, and until the insured persons have been allocated there is no means of distributing the fund in question. The fundamental basis of the remuneration of the doctors on a capitation system is that they receive the prescribed allowance for insured persons on their list whether they are ill or well. While only those who are ill take the trouble to place their names on the doctors' lists, it is clear that the doctors are obtaining as patients only those to whom they have to give immediate attendance, and in consequence the remuneration is inadequate without the allocation of those insured persons who have not themselves chosen a doctor.

It is evident, therefore, that service on the Insurance Committees is a matter that requires expert administrators, and yet the regulations of the Insurance Commissioners governing the election of the representatives of insured persons have been allowed to differ essentially from each other. There can surely be no reason why such important matters should not be given adequate discussion, and that a single comprehensive scheme equally applicable to all countries of the Union should be devised. There has been too much experimental dealing with important matters in connection with the National Insurance Act, and far too little clear thinking and planning in advance.

It was so in regard to the institution of the four separate Commissions. The original Bill made no reference to such divided authority, and the separate Commissions were the result of an amendment passed almost without discussion, and certainly, as it seems to us, without any clear comprehension by the framers of the Act of the administrative confusion which has resulted therefrom.

### Transfers and Valuations.

As a consequence of the institution of the four Commissions it has become necessary for continual transfers to be made in the books of approved societies having members in more than one country each time members removed from one country to another. This process is troublesome, and, so far as the societies and insured persons are concerned, it is a useless waste of labour. The worst results of the present system will not become apparent until the first actuarial valuations are made. Upon such a valuation the liabilities of the society to its members in each country will have to be separately investigated, and any surplus that may be available in any one country for the provision of additional benefits will have to be rigorously applied in that country only, just as though the members belonged to an independent society. Furthermore, it may be that on account of the membership of the society in one country being less than 5,000, the members will have to be grouped with those of other societies and perhaps share in a deficit and consequent reduction of benefits, while members of the same society across the border are able to obtain increased benefits as the result of the careful management of the society as a whole. This is more than a mere possibility, and, quite apart from the extra labour and expense involved by the four sets of Commissioners, it is evident that grave administrative difficulties are likely to arise unless some amendment of the position is speedily secured.

### Unification of Control.

It may be necessary for the separate clerical staffs to continue their work in London, Edinburgh, Dublin, and Cardiff respectively, but the national character of the Act should, in our opinion, be preserved by the immediate abolition of the four sets of Commissioners and the institution of a single body, better paid if necessary, but smaller in size, which could undertake the comprehensive control of the administrative machinery for the whole of the United Kingdom.

The amending Bill is understood to be almost ready for introduction to Parliament, and the Government is bound to be faced with amendments which it cannot accept. It is, however, on the basis of experience that the Act is to be amended, and experience has conclusively shown that divided authority is wasteful and unnecessary.

### Answers to Correspondents.

### A Nurse's Liability to Become Insured.

Nurse S. F. B. informs us that she is a private nurse, most of her work being maternity cases, and she asks if she has to be insured under the National Insurance Act.

We cannot do better than refer her to our issue of August 10 last (p. 486), in which a letter from the National Health Insurance Commission (England) is given. The Commissioners there state that "nurses working on their own account will be required to be insured, and the persons by whom they are engaged will, as the employers, be liable for the payment of the contribution. The contributions due from the nurse will be recoverable by deduction from her remuneration. . . The only case, generally, in which a nurse will not have to be insured (subject to the ordinary provisions of the Act as to casual employment and employment at a rate of remuneration exceeding in value £160 a year) is the case where she receives patients into her own house for treatment."

### The Disposition of Sickness Benefit.

We are also asked by the secretary of a hospital if we have any knowledge of the practice of hospitals in regard to the disposition of sickness benefit received by nurses when treated in hospital. We publish in another column the course to be adopted in the case of medical benefit by the London Hospital authorities. In regard to sickness benefit the plan to be followed is to deduct 7s. 6d. a week during each week's illness from the nurse's salary. This ensures the recovery of sickness benefit from the approved society.

Employers other than hospital authorities pay full remuneration to their employees when they are ill, and thus become entitled under Section 47 of the Act to a reduction in the weekly contributions if they will guarantee the payment of full salary for the first six weeks of sickness. Many employers are taking advantage of this section; but others, while continuing the practice of paying full remuneration to their employees when they are ill, are paying the full rate of contributions, and are obtaining the advantage of those payments by an amicable arrangement with the employees whereby the latter receive full salary, less the sickness benefit, when they are ill. It is probably safe to say that the majority of employers are adopting the latter course. It may, however, be pointed out that any attempt to give legal effect to such deductions from salary could not be upheld, for it is expressly provided by Section 111 that "every assignment of, or charge on, and every agreement to assign or charge any of the benefits conferred by the Act shall be void."

**NOTE.—Questions and Correspondence on all doubtful points are invited, and should be addressed to the Editor, The Hospital, 28, 29 Southampton Street, Strand, London, W.C., and marked "Insurance."**

